# Supplementary material for: Exploring the implementation of community health worker program in Fiji: An exploratory qualitative study
Source: PLOS Glob Public Health. 2025 Dec 23;5(12):e0005583. doi: 10.1371/journal.pgph.0005583 (PMC12725549; doi:10.1371/journal.pgph.0005583)
Supplement: S1 Appendix — (DOCX) [file pgph.0005583.s001.docx]

**S1 Appendix. Characteristics of the FGD participants**

| **Participant number** | **Sex** | **Years Worked as a CHW** | **Age** | **Division** | **Highest Level of Education** |
| --- | --- | --- | --- | --- | --- |
| P1 | F | <2 years | 39 | Eastern | Secondary |
| P2 | F | >6 years | 49 | Eastern | Secondary |
| P3 | F | 2-6 years | 52 | Eastern | Secondary |
| P4 | F | >6 years | 55 | Eastern | Secondary |
| P5 | F | 2-6 years | 65 | Eastern | Secondary |
| P6 | F | <2 years | 43 | Eastern | College |
| P7 | F | >6 years | 50 | Eastern | Primary |
| P8 | F | 2-6 years | 55 | Eastern | Secondary |
| P9 | F | >6 years | 45 | Western | College |
| P10 | F | 2-6 years | 51 | Western | College |
| P11 | M | <2 years | 48 | Western | Secondary |
| P12 | F | >6 years | 53 | Western | Secondary |
| P13 | F | >6 years | 49 | Western | College |
| P14 | M | <2 years | 36 | Western | Secondary |
| P15 | F | >6 years | 47 | Western | Secondary |
| P16 | F | 2-6 years | 30 | Western | Secondary |
| P17 | F | >6 years | 57 | Central | Secondary |
| P18 | F | >6 years | 42 | Central | Secondary |
| P19 | F | >6 years | 51 | Central | Secondary |
| P20 | F | <2 years | 37 | Central | Secondary |
| P21 | F | >6 years | 41 | Central | Postgraduate |
| P22 | F | <2 years | 37 | Northern | College |
| P23 | F | >6 years | 41 | Northern | Secondary |
| P24 | F | 2-6 years | 49 | Northern | Secondary |
| P25 | F | >6 years | 37 | Northern | College |
| P26 | F | >6 years | 53 | Northern | Primary |
| P27 | F | 2-6 years | 39 | Northern | College |
| P28 | F | >6 years | 45 | Northern | Secondary |
| P29 | F | <2 years | 40 | Northern | Secondary |
| P30 | F | >6 years | 60 | Northern | Secondary |
| P31 | F | >6 years | 51 | Central | Secondary |
| P32 | F | >6 years | 44 | Central | Secondary |
| P33 | F | >6 years | 46 | Western | Secondary |
| P34 | M | 2-6 years | 51 | Western | Secondary |
| P35 | F | >6 years | 49 | Western | College |
| P36 | F | <2 years | 53 | Western | Secondary |
| P37 | F | 2-6 years | 53 | Western | College |
| P38 | F | <2 years | 38 | Western | Secondary |
| P39 | F | >6 years | 50 | Western | College |
| P40 | F | 2-6 years | 43 | Western | Secondary |
| P41 | F | >6 years | 60 | Western | Secondary |
