# Supplementary material for: Exploring the implementation of community health worker program in Fiji: An exploratory qualitative study
Source: PLOS Glob Public Health. 2025 Dec 23;5(12):e0005583. doi: 10.1371/journal.pgph.0005583 (PMC12725549; doi:10.1371/journal.pgph.0005583)
Supplement: S1 File — (PDF) [file pgph.0005583.s002.pdf]

Interview guide (Policy makers / Supervisors)

Aim of the interview

This interview is part of a research study "Assessing the role of community health workers in Primary Health Care in Fiji".

EXPLORATORY QEUSTIONS:

1. Role of CHWs overall and for CVD service delivery

Can you please tell us about the role of the CHWs? Is there any available guidelines for their role?

How do the CHWs get introduced to their job role?

How do the CHWs get feedback regarding their role? Any guidance?

Is their role and task(s) clearly understood within the community?

How do you think the PEN program can be introduced to the CHW program?

2. Training

Can you please tell us more about the available training provided to CHWs?

Any special guidelines for the NCD management training?

How are they trained and evaluated? When PEN is integrated into the CHW program, how will the CHWs be trained to deliver quality health care?

Can you please tell us about the continuous training of the CHWs, are all the CHWs included or do you follow special guidelines to select the CHWs for training?

3. Equipment and Supplies

Can you please tell us more about the equipment and supplies provided for the CHWs?

As MHMS is planning to roll out PEN, how will the equipment and infrastructure be maintained to deliver care for CVD?

4. Supervision

Can you please tell us about the supervision of CHWs? What is the process for supportive supervision?

Can you tell us about supervision checklists or any other supervision tools that exist to help guide supervisors?

5. **Individual Performance Evaluation**

Can you please tell us about the process for conducting individual performance evaluations for CHWs? Who is usually involved in the evaluation process?

6. **Incentives**

Can you please tell us about the available guidelines for the financial or non-financial incentives provided to CHWs? Are these guidelines based on performance evaluation? Are there any extra incentives for the NCD management role?

7. **Referral System**

Can you please tell us about the referral system provided by the CHWs? As CHWs identify people with diabetes and hypertension, what are the plans for the referral to the local PHC/doctor and follow up of patients in the community?

What are the guidelines followed by the CHWs to determine when a referral is needed?

What are the followed guidelines for follow-up on these referrals? How does the health centre provide CHWs with information about the patient once the referral has been made?

What are the current established logistics plan for emergencies in the community?

8. **Opportunity for Advancement/ CAREER PROGRESSION**

Can you please tell us about the available advancement opportunities for CHWs?

How do the CHWs usually get informed about these opportunities?

9. **Community PEN Specific Questions**

- Do the current modules for training include NCD training for CHWs?
- How do CHWs look after people with NCDs living in their community – how do they support them?
- Do you feel that CHWs have the capacity, skills and equipment to look after patients with NCDs in their community?
- If yes then how has this been supported or can be supported?
- If no – then what capacity, equipment and skills do they need?
- Does your scope of practice allow them to look after patients with NCDs and if not then what changes are required?

– Would they be willing to look after people with NCDs in their community and would the department support this?

10. Documentation and Information Management

Can you please tell us about the documentation system followed by the CHWs? How do they keep records of their home visits and other services provided? Are these paper based? How do supervisors monitor the quality of documents and provide assistance when needed? Do the data they collect form part of the health information system?

11. Program Performance Evaluation

What is the available systematic process for conducting program performance evaluations for the whole CHW program? What is the evaluation based on? program targets, objectives, or indicators? What is the current process to provide feedback to CHWs based on the evaluation?
