# Supplementary material for: Exploring the implementation of community health worker program in Fiji: An exploratory qualitative study
Source: PLOS Glob Public Health. 2025 Dec 23;5(12):e0005583. doi: 10.1371/journal.pgph.0005583 (PMC12725549; doi:10.1371/journal.pgph.0005583)
Supplement: S2 File — (PDF) [file pgph.0005583.s003.pdf]

CHW FGDs guide

The aim of the meeting

This Focus Group discussion will be conducted to understand the current role of the CHWs in delivering primary health care to the community. We would like to know more about the activities that you perform and the challenges that you face in delivering care.

1. Recruitment and role of CHWs

- How were you recruited to be CHW? (Community, government, NGO/CBO, Other (please explain)
- How were you assigned to the community(s) in which you currently work?
- Tell us about your current role as a CHWs? What are your responsibilities?
- Can you tell us about your experience in providing services for the community?
- What are the main challenges that you face while delivering health care, support and awareness?
- Did you talk to patients about their lifestyle risk factors? If so how did they respond to your advice?

2. Training

- Did you receive any initial training to prepare you for your role? Were you evaluated before or after this training?
- Have you been trained for chronic diseases management?
- A new program for hypertension and diabetes control is being launched, how do you think you can contribute towards this role.
- Do you usually receive any additional training (refresher/ongoing training) to help you fulfil your role? Do you get evaluated after receiving training?
- Who do you contact if something is unclear to you while doing your job or if you encounter any challenges in the community?

3. Equipment and Supplies

— Do you have the supplies and equipment you need to provide the services you are expected to deliver?

— Do you dispense any medicines? If you have experienced shortages or stock outs of supplies, how do you get more supplies?

— Probe: How often do you get them? / Do you use any form(s)? / How do you get the form(s) and to whom do you submit them?

4. **Supervision**

— Could you tell us about the formal supervisory mechanism? How often does your supervisor visit you?

— What does your supervisor do when he/she visits you? Probe: Observation of service delivery, Coaching and skills development, Trouble shooting, problem solving, Record Review, Supply check

— Do they usually accompany you in field visits or you meet them at the health centre?

— Does your supervisor provide you any feedback regarding your performance?

5. **Individual Performance Evaluation and incentives**

— Have you received a formal evaluation of your work in the last 12 months? Probe: Could you please explain the process of evaluation?

— Do You receive any feedback regarding this evaluation?

— Do you receive any payments for your role?

— Is it based on specific tasks?

6. **Community Involvement**

— Does the community provide any support to you? How? Probe: Feedback, Support (financial/gifts in kind), Formal recognition/appreciation, Guidance on your work

— How did the patients respond to the chronic disease care provided to them in their houses?

— How did they respond to the results of their BP, blood sugar?

7. **<u>Referral System</u>**

- How do you refer clients for health services you do not or cannot provide?
- What about chronic diseases, how does their referral system work?

8. **<u>Opportunity for Advancement/ Career progression</u>**

- What are your opportunities for promotion or professional advancement through the CHW program?

9. **<u>Community PEN Specific Questions</u>**

- Are you aware of what non-communicable diseases are?
- Do you have any people with NCDs living in your community – how do you support them?
- Do you feel that you have the capacity, skills and equipment to look after patients with NCDs in your community?
- If yes then how has this been provided
- If no – then what capacity, equipment and skills do you need?
- Does your scope of practice allow you to look after patients with NCDs and if not then what changes are required?
- Would you be willing to look after people with NCDs in your community?

10. **<u>Documentation and Information Management</u>**

- How do you keep records of all your patients? Do you have a patient register?
- Tell me about the CHW report, how often do you complete the report and who do you send it to? Do you record all services provided?
- How are your records linked to your supervisors or the health system?
-

<u>End of session questions</u>

- Is there anything else that you would like to share with me?
