# Supplementary material for: Exploring the implementation of community health worker program in Fiji: An exploratory qualitative study
Source: PLOS Glob Public Health. 2025 Dec 23;5(12):e0005583. doi: 10.1371/journal.pgph.0005583 (PMC12725549; doi:10.1371/journal.pgph.0005583)
Supplement: Attachment — Submitted filename: Response to Reviewers_CHW program Fiji.docx [file pgph.0005583.s007.docx]

19 November 2025

**Author Response Letter: PLOS Global Public Health**

**Evaluation of community health worker program in Fiji: A qualitative exploratory study [PGPH-D-25-01870]**

Dear Dr Gemma Lea Saravanos, Academic Editor

I am writing on behalf of all authors to submit the revised manuscript on “*Evaluation of community health worker program in Fiji: A qualitative exploratory study*”. We appreciate the opportunity to respond to the reviewers’ comments and have prepared a point-by-point response to the editorial and reviewers’ comments below. Clean and tracked change manuscripts are also submitted per the Journal’s requirements.

**Journal Requirements:**

1. Please include a complete copy of PLOS’ questionnaire on inclusivity in global research in your revised manuscript. Our policy for research in this area aims to improve transparency in the reporting of research performed outside of researchers’ own country or community. The policy applies to researchers who have travelled to a different country to conduct research, research with Indigenous populations or their lands, and research on cultural artefacts. The questionnaire can also be requested at the journal’s discretion for any other submissions, even if these conditions are not met. Please find more information on the policy and a link to download a blank copy of the questionnaire here: <https://journals.plos.org/globalpublichealth/s/best-practices-in-research-reporting> Please upload a completed version of your questionnaire as Supporting Information when you resubmit your manuscript.

**Response:** Thank you. We completed the questionnaire and submitted as supporting information with the revised manuscript. We have also added a sub-section on Inclusivity in global research in the methods section.

2. If the reviewer comments include a recommendation to cite specific previously published works, please review and evaluate these publications to determine whether they are relevant and should be cited. There is no requirement to cite these works unless the editor has indicated otherwise.

**Response:** Thank you for the suggestion. The reviewers did not recommend any specific publications to be cited in this manuscript.

**Reviewers’ comments:**

**Reviewer 1:**

Congratulations to the authors on the efforts and such a well-written article. The original data, albeit qualitative only, from Fiji are helpful to understand the issues/barries/enablers of the CHW program. The authors have also considered regional disparities and purposively selected interviewees from the four divisions of Fiji.

**Response:** Thank you for your words of support.

1. Program evaluation usually has baseline assessment and evaluates how the outcomes of interest have changed due to the implementation of the program, via comparing with the baseline. Does this study have any baseline assessment? Or technically speaking, this study is not an evaluation of the CHW program but only identifies current issues/barriers/enablers of the CHW program.

**Response:** We thank the reviewer for the valuable comment. The study did not include a baseline assessment, and we acknowledge that traditional program evaluations typically measure changes against baseline data. Hence, the study title has been revised to better reflect the nature of the work as an exploration of the implementation of the CHW Program in Fiji rather than an outcome evaluation. Please refer to the title of the revised manuscript (track changed manuscript).

2. It seems unclear how the authors determined the sample size, and the justifications behind the selection of the key informants.

**Response:** The sample size was guided by the principle of data saturation, with interviews continuing until no new themes emerged. Key informants were purposively selected from each of the six sub-divisions to include participants with direct experience and knowledge of CHW program implementation, ensuring information-rich perspectives relevant to the study objectives. The Methods section has been revised to clarify these points. Please refer to Page 5, Lines 14-24 of the revised manuscript.

3. Were two independent researchers involved to generate themes? What did the study team do to ensure the results/themes summarised were unbiased?

**Response:** Initial coding of the interview transcripts was conducted by one author. To enhance credibility and trustworthiness, emerging codes and themes were then discussed and reviewed iteratively by the study team. Team discussions ensured that the final themes accurately reflected the data. We have clarified this point in the revised manuscript (Page 6, lines 13-26).

4. The authors may need to add age and region of the respondent of the quotes.

**Response:** We appreciate this important suggestion from the reviewer. Detailed demographic and professional characteristics of the FGD participants (CHWs) are now provided in Appendix 1. However, we have intentionally not included the age and sub-divisional details of key informant interview participants to protect confidentiality and prevent potential identification, given their small number and specific positions within the ministry.

5. Is there any analysis regarding how the issues/barriers/enablers identified are similar or different across regions, and what causes the difference, if any?

**Response:** Yes, we conducted an analysis to explore this. During thematic analysis, we identified similarities and differences in CHW program implementation, barriers and enablers across divisions, which were highlighted within the eight main themes. Contextual factors contributing to these variations are also discussed where relevant. We appreciate the reviewer’ requests for clarification.

6. The discussion section is too long. Although the authors tried to mention every aspect based on the framework, a more structured organisation might help better highlight the key messages: eg. summary of key findings, key barriers and facilitators of the CHW program identified through the interviews, policy recommendations, strengths and limitations etc.

**Response:** We thank the reviewer for this helpful suggestion. We have shortened the Discussion to improve clarity and better highlight the key messages. Please refer to the Discussion section of the revised manuscript.

7. Authors need to expand the limitations. For example, this study design, i.e. qualitative interview with key informants only, has largely limited the interpretation and extrapolation of the results. This is just an explorative study, and more robust design using mixed methods is needed in the future to evaluate the CHW program.

**Response:** We thank the reviewer for this comment. We have expanded the limitations section to note that the qualitative design limits interpretation and generalisability, and the absence of community perspectives further restricts insights to the provider side. We also highlight that future evaluations should use more robust mixed methods designs to comprehensively assess the CHW program.

**Reviewer 2:**

The topic of community health workers (CHWs) in Fiji is highly relevant and timely, particularly in the context of rural healthcare delivery and ongoing health system challenges in Fiji. The manuscript covers important ground; however, there are several areas where clarity and depth could be improved to strengthen the overall contribution of your study. I provide some suggestions below, which I hope will help you strengthen your manuscript.

1. Introduction: The introduction would benefit from a clearer and more explicit definition of CHWs upfront. This should include their roles, responsibilities, the population they serve, and the scope of medical care they are authorised to provide. Establishing this foundational understanding will better contextualise your findings and allow the reader to assess the alignment, or lack thereof, between CHW responsibilities and actual practice as described in your results.

**Response:** We thank the reviewer for this important suggestion. We have revised the Introduction to provide a clearer and more explicit definition of CHWs in the Fijian context. The revised text now includes their selection criteria, roles, responsibilities, and the population they serve, as well as the scope of care they are authorised to provide according to the Fiji Ministry of Health and Medical Services CHW policy. Please refer to Page 3, line 13-18.

2. Please clarify whether there is a difference between CHWs and village health workers in the Fijian context. If so, this should be explicitly stated.

**Response:** We thank the reviewer for this important point. In the Fijian context, CHWs are locally recruited and stationed within communities and are broadly similar to village health workers in other settings. There is no separate cadre called “village health workers” in Fiji. We have clarified this in the second paragraph of the Introduction section of the revised manuscript. Please refer to Page 3, line 13-18.

3. Ensure all acronyms are spelled out at their first instance.

**Response:** We ensured that all acronyms are spelled out at their first instance. A few acronyms (such as National Community Health Workers Steering Committee (NCHWSC) and Ministry of Health and Medical Services (MOHMS) have also been expanded in subsequent sections to enhance readability and clarity.

4. You reference four previous studies assessing Fiji’s CHW program. It would strengthen your argument to articulate how your study builds upon or differs from this existing literature. What is the unique contribution of your study, and why was it necessary?

**Response:** We thank the reviewer for this valuable suggestion. We have revised the last paragraph of the Introduction section to clarify the focus of the four previous studies and to highlight how our study uniquely contributes to the existing body of knowledge by emphasizing its comprehensive scope. Please refer to Page 4, lines 3–12 in the revised manuscript.

5. Consider providing a brief discussion of the current health workforce challenges in Fiji in the introduction. This would help set the stage for understanding the importance and reliance on CHWs, particularly in rural and remote communities.

**Response:** A brief sentence has been added in the Introduction noting health workforce shortages in rural and remote areas of Fiji, providing context for the critical role of CHWs in supporting primary health care delivery and linking communities with the formal health system. Please refer to the Page 3, lines 6-8 of the revised manuscript.

6. Note: the official country name is "the Republic of Fiji".

**Response:** We appreciate the reviewer’s observation regarding the official country name. While “the Republic of Fiji” is the formal designation, we have confirmed that both national and WHO reports commonly use “Fiji”. In consultation with co-authors from Fiji, we prefer to retain “Fiji” throughout the manuscript for consistency with these sources.

7. Methods: The methods section could be strengthened with more detail. For example:

- Was a theoretical framework used to inform or underpin the study design?
- How was the co-design with stakeholders operationalised in practice?
- What were the inclusion/exclusion criteria for participant selection?
- How were participants approached and recruited for the study?
- What experience or background did the research team have in conducting qualitative interviews?

**Response:** We thank the reviewer for these comments. We have revised the Methods section to provide additional clarity and detail on each point.

- Theoretical framework: The Community Health Worker Assessment and Improvement Matrix (CHW AIM) framework was used to guide the development of the data collection tools and analysis (Page 6, lines 17-19).
- Co-design with stakeholders: We have clarified how the co-design process was operationalised. Preliminary findings were presented to stakeholders during a workshop where participants validate the findings and provided contextual feedback, which the research team incorporated into the final manuscript (Page 6, lines 21–24).
- Inclusion and exclusion criteria: The inclusion and exclusion criteria for participant selection have been revised for clarity and are now clearly presented in the Methods section (Page 5, lines 14–24).
- Participant approach and recruitment: We provide additional detail on recruitment procedures. Participant selection was conducted with the assistance of the Chief Nursing and Midwifery Officer, who communicated with nurse managers. The in-country research team then coordinated the interviews. CHWs were contacted through their respective nurse managers and FGDs were arranged in a similar manner (Page 5, lines 14-24).
- Research team experience: The team (RJ, AGT, BP) has extensive experience conducting qualitative research across diverse settings and has published multiple peer-reviewed papers using qualitative methods.

8. Please include a reference for the coding approach used in your qualitative analysis.

**Response:** We have now cited Fereday & Muir‑Cochrane (2006) (Ref # 21), to support our abductive coding approach, which iterates between inductive and deductive processes to generate codes and develop themes.

9. A demographic table summarising participant characteristic would provide helpful context.

**Response:** Thank you for this valuable suggestion. As noted in our response to Reviewer 1 (#4), demographic and professional details of FGD participants have been added in Appendix 1. To maintain confidentiality, we have not included age or sub-divisional identifiers for key informant interview participants due to their small number and identifiable roles.

10. Results: Several claims throughout the findings would benefit from more context or supporting evidence:
    10.1. In Theme 1, how was the assessment made that CHWs in the Central Division were better trained and more engaged?

**Response:** We thank the reviewer for these important questions on the results section. In Theme 1: The assessment was based on participants’ accounts during the interviews and FGDs. Supervisors and CHWs from the Central and other divisions consistently described more frequent training opportunities and more engagement of CHWs in community activities compared with other divisions. We have highlighted similar comparisons across the relevant themes in the Results section. We also revised the results in Theme 1 to clarify this, please refer Page 7. Lines 27-30.

10.2. In Theme 3, further detail is needed on the nature of the training received by newly enrolled CHWs if it does not meet requirements.

**Response:** We have expanded Theme 3 to provide additional detail on the training received by newly enrolled CHWs. Participants reported that recent recruits often received only short, one-week trainings, with variations in content, which fell short of the comprehensive six-week program previously offered. These differences have been clarified in Themes 3 and 1 of the Results section.

10.3. In Theme 5, you state that CHWs with strong relationships with zone nurses felt empowered, but the accompanying quote lacks sufficient context to support this. Consider expanding on the quote or including an additional quote that illustrates the sense of empowerment more clearly.

**Response:** We have revised Theme 5 to provide additional context on supervisor support. An additional quote from another FGD has been included to illustrate how supportive interactions with zone nurses enabled CHWs to perform their duties and seek guidance when needed. Please refer to Page 11 end and beginning of Page 12.

10.4. Clarify what is meant by “regular visits by higher-level bodies” in Theme 5—are these supervisors, Ministry of Health officials, or others?

**Response:** We have clarified Theme 5 to specify that ‘regular visits by higher-level bodies’ refer to supervisory visits from sub-divisional nurse managers and medical officers (Page 12, lines 27–29).

10.5. In Theme 6, the phrase “sense of service” is vague and may benefit from rephrasing or further elaboration.

**Response:** We have revised the sentence in Theme 6 to elaborate on the phrase “sense of service,” refers to CHWs’ feeling of responsibility and commitment to serving their communities and addressing the health needs of their people (Page 14, lines 1-3).

10.6. In Theme 7, specify what basic supplies CHWs are purchasing out-of-pocket and what they are officially meant to be provided with. Also, the quote from Participant 03 suggests CHWs distribute a range of medications. Earlier in the manuscript, it was implied that CHWs are not authorised to dispense medication. This discrepancy requires clarification or commentary.

**Response:** We have clarified Theme 7 to specify that CHWs often purchase basic supplies (e.g., gloves, dressings, paracetamol, and ORS) out-of-pocket, which are officially provided. We also clarified that CHWs are not authorised to dispense prescription medications according to policy (Page 14, lines 8-16).

10.7. Aim for consistency in phrasing throughout e.g., use either “village headman” or “village heads” consistently.

**Response:** We carefully reviewed the Results section and ensured consistent use of the phrase “village headman” throughout the manuscript.

10.8. Ensure all quotes are attributed to specific participants using participant numbers consistently.

**Response:** We have carefully reviewed the manuscript and ensured that all quotes are consistently attributed to specific participants or, in the case of FGDs, to the relevant group using their assigned participant numbers. For FGDs, the attributions are as follows: Eastern Division (Levuka: FGD-07), Northern Division (Seaqaqa: FGD-03), Central Division (Nausori: FGD-04; Valelevu: FGD-05), and Western Division (Balevuto: FGD-01; Sigatoka: FGD-06). For the six supervisors and nursing managers, participants are labeled as Supervisor (S01–S06).

10.9. You may wish to reflect on the use of social media by CHWs for communicating with supervisors, and whether this presents any risks related to data privacy or confidentiality.

**Response:** We thank the reviewer for this suggestion. We have added a note in the Discussion highlighting that CHWs’ use of social media with supervisors may pose data privacy and confidentiality risks and emphasized the need for guidance on secure communication (Page 17, lines 15-18).

11. Discussion
    11.1. In the first paragraph, you state that "we observed……a strong community trust in the CHW program.” This claim is not clearly substantiated in the results section and should either be supported with data or rephrased.

**Response:** We thank the reviewer for this comment. As our study did not include community perspectives and the findings are based solely on CHWs’ reports, we have rephrased this statement in the Results and Discussion sections to reflect that it represents CHWs’ perceptions of community trust rather than direct evidence from the community. Please refer to Theme 8 (Results: Page 15, lines 11-12) and in the Discussion: Page 16, lines 4–6.

11.2. Consider softening definitive statements such as “the Ministry needs to empower…” to more measured language like “empowering the local level of the health system may support…”

**Response:** We thank the reviewer for this suggestion. We have revised the manuscript to use more measured language, as recommended. Please refer to Page 16, line 28-32.

12. The limitations section needs further development to clearly acknowledge the study’s methodological constraints.

**Response:** Thank you for the comment. As noted in our response to Reviewer 1, comment #7, we have expanded the Limitations section to include the study’s methodological limitations and suggestions for future improvements.

Thank you again for your feedback and for considering our revised paper. Please do not hesitate to contact me if you have any further questions.

Best regards,

Azeb Gebresilassie Tesema
