# Supplementary material for: Exploring the implementation of community health worker program in Fiji: An exploratory qualitative study
Source: PLOS Glob Public Health. 2025 Dec 23;5(12):e0005583. doi: 10.1371/journal.pgph.0005583 (PMC12725549; doi:10.1371/journal.pgph.0005583)
Supplement: Attachment — Submitted filename: Response to Reviewers.docx [file pgph.0005583.s008.docx]

3 December 2025

**Author Response Letter: PLOS Global Public Health**

**Exploring the implementation of community health worker program in Fiji: An exploratory qualitative study [PGPH-D-25-01870R1]**

Dear Dr Gemma Lea Saravanos, Academic Editor

On behalf of all authors, I would like to thank you and the reviewers for evaluating our revised manuscript and for your positive acknowledgment of our efforts to address the comments. We have incorporated all revisions suggested by you and the reviewers. Please find below our point-by-point responses to the comments. Both the clean and tracked-changes versions of the manuscript have been submitted in accordance with the journal’s requirements.

**Comments**

**Methods:**
**1. Page 5, line 22** you state that “Six FGDs with 41 CHW and the same number of key informant interviews with divisional nurse supervisors and divisional managers were conducted across six subdivisions representing Fiji’s four administrative divisions"
It is not clear to me how many key informant interviews were conducted and with whom and why. Please explicitly state how many key informant interviews were conducted, who was interviewed and how this informed your results. You may need to describe this in a separate sentence so that your methods are clear. This detail (in brief) should also be included in the abstract.

**Response:** Thank you for this important suggestion. We agree that the description of the key informant interviews required clarification. We have now explicitly stated the number of interviews conducted, who was interviewed, and how these interviews contributed to the analysis.

In the revised manuscript, we clarified that we conducted six key informant interviews with CHW supervisors and nursing managers across the selected subdivisions. We added a separate sentence in the Methods section explaining the purpose of selecting these key informants and how their perspectives informed the interpretation of the study. We have also included a brief information in the Abstract, as recommended. Additionally, to make the key informant participant codes clearer in the results quotes, we have revised them from S01–S06 to KII01–KII06. Please refer to the Methods section on page 5 and the Results section on page 7 of the revised manuscript (tracked changes version).

**Results:**
**2**. Page7, line 13 “mangers” should read “managers”

**Response:** We appreciate your careful reading and have corrected the typo to “manager”. Please refer to Page 7, lines 16-18 in the revised manuscript.

**3.** Page 8, line 4 “headmens” should read “headmans”

**Response:** Thank you for the suggestion. We could not find the word “headmens” in the manuscript; however, we have revised the sentence in the same page “We noted between-area variations in the support and involvement of the village headmen” to use the singular form “headman.”

**Discussion:**
**4.** Page 18, line 19 you state that the process must consider variations in geographical divisions as well as the impact of COVID-19 on the health system, however the impact of COVID-19 has not been discussed elsewhere in your manuscript, and it is not clear how this is relevant to your study. Please clearly describe the specific impact of the COVID-19 pandemic and how it is relevant to the standardisation of CHW’s roles, and why this must be considered. Else consider removing this from your discussion.

**Response:** Thank you for the suggestion. We have removed the discussion of the impact of COVID-19 on the health system from the manuscript.

**Appendix 1:**
**5**. Thank you for providing this detailed description of FGD participants. Please consider if age as you have provided presents any privacy concerns, and report age brackets if necessary.

**Response:** We appreciate the suggestion regarding potential privacy concerns for FGD participants. The research team discussed this issue and agreed that the current age data provided for the FGD participants do not pose a privacy risk, as there are multiple CHWs in each subdivision with similar age groups.

Journal Requirements:

Please review your reference list to ensure that it is complete and correct. If you have cited papers that have been retracted, please include the rationale for doing so in the manuscript text, or remove these references and replace them with relevant current references. Any changes to the reference list should be mentioned in the rebuttal letter that accompanies your revised manuscript. If you need to cite a retracted article, indicate the article’s retracted status in the References list and also include a citation and full reference for the retraction notice.

**Response:** We have carefully reviewed the reference list to ensure that it is complete and accurate. No retracted articles were cited.

Thank you again for your feedback and for considering our revised paper. Please do not hesitate to contact me if you have any further questions.

Best regards,

Azeb Gebresilassie Tesema
